# Predictive value of S100A4 in eosinophilic chronic rhinosinusitis with nasal polyps

**DOI:** 10.3389/fsurg.2022.989489

**Published:** 2022-11-01

**Authors:** Shangchu Zou, Zhicheng Huang, Jinpeng Wu

**Affiliations:** ^1^ The Affiliated Nanhua Hospital, Department of Otolaryngology Head and Neck Surgery, Hengyang Medical School, The University of South China, Hengyang, China; ^2^ The Second Affiliated Hospital, Department of Otolaryngology Head and Neck Surgery, Hengyang Medical School, University of South China, Hengyang, China; ^3^ The First Affiliated Hospital, Department of Otorhinolaryngology Head and Neck Surgery, Xiamen University, Xiamen, China

**Keywords:** s100A4, eosinophilic, chronic rhinosinusitis with nasal polyps (CRSwNP), predictive, endotype

## Abstract

**Objective:**

S100A4 is a pro-inflammatory mediator which has been implicated in airway inflammatory diseases. However, its role in chronic rhinosinusitis with nasal polyps (CRSwNP) remains unclear. The purpose of this study is to determine the expression of S100A4 and evaluate its potential value in distinguishing its endotypes.

**Methods:**

Sixty CRSwNP patients, 30 chronic rhinosinusitis without nasal polyps (CRSsNP) patients, and 30 healthy controls (HC) were enrolled in this study, and serum and tissue samples were collected. Serum and tissue S100A4 levels were detected by enzyme-linked immunosorbent assay, reverse transcription-polymerase chain reaction, western blotting and immunofluorescence. Their clinical values in predicting postoperative recurrence of CRSwNP were evaluated by multivariate analysis and ROC curves.

**Results:**

Serum levels of S100A4 were notably increased in the CRSwNP group than in the CRSsNP and HC groups (*p* < 0.05), and positively correlated with tissue and peripheral eosinophil count and percentage (*p* < 0.05). The serum S100A4 concentrations were significantly elevated in the Eos CRSwNP group in comparison with the non-Eos CRSwNP group (*p* < 0.05). Multivariate analysis and ROC curve presented that serum S100A4 levels were associated with CRSwNP endotypes. Additionally, tissue S100A4 mRNA and protein levels were significantly enhanced in the CRSwNP group than in the HC group and CRSsNP group, especially in the Eos CRSwNP group.

**Conclusion:**

Our results demonstrated that the S100A4 expression was increased in CRSwNP patients and associated with the endotypes. S100A4 could be a serologic biomarker for evaluating tissue eosinophilic inflammation and predicting endotypes in CRSwNP patients.

## Introduction

Chronic rhinosinusitis (CRS) is a highly heterogeneous inflammatory disorder and affects a large portion of the global population (1, 2). CRS is frequently categorized into 2 major phenotypes: CRS with nasal polyps (CRSwNP) and CRS without nasal polyps (CRSsNP). CRSwNP is primarily characterized by type 2 inflammation with a large accumulation of inflammatory cells, which is dominated by eosinophils (3, 4). Moreover, the degree of eosinophil infiltration in CRSwNP has a vital impact on the clinical characteristics, the timing of surgery, drug efficacy, and prognosis (5). Therefore, it has been proposed that CRSwNP could be grouped into two endotypes based on the degree of infiltration of eosinophils into the nasal mucosa: eosinophilic CRSwNP (Eos CRSwNP) and non-eosinophilic CRSwNP (non-Eos CRSwNP) (6, 7). These two endotypes present different cellular and pathophysiological characteristics, clinical manifestations and treatment outcomes, and Eos CRSwNP exhibits a high symptom burden and poor quality of life due to its severe clinical conditions and recurrence (8). Although the visual analog scale (VAS) and Lund-Kennedy endoscopy score, and total nasal symptom score (TNSS) score have been used to assess the severity of the disease (9, 10), these methods were not objective sufficiently. Therefore, it is crucial to identify potential objective biomarkers to assess disease severity and differentiate the endotypes of CRSwNP for achieving precise treatment.

S100A4 is a member of the S100 calcium-binding protein family, and it is expressed in a variety of cell types of lymphoid and myeloid lineages (11, 12). S100A4 was proven to be involved in the pathophysiology of fibrotic, inflammatory, and autoimmune disorders and induced and promoted tumor metastasis (13, 14). Moreover, S100A4 could induce the expression and secretion of pro-inflammatory cytokines and stimulate the activation of different processes by pro-inflammatory-related pathways (14). Furthermore, a previous study found that S100A4 could alter the polarization balance of T cells toward the Th2 phenotype, suggesting that S100A4 could affect T cell differentiation (15). A recent study observed that S100A4 expression was increased in the serum of allergic rhinitis patients, and s100A4 gene knockout presented a protective effect against allergic inflammation in animal models, suggesting that S100A4 might be closely involved in airway inflammation (16). Thus, this study aimed to explore the role of S100A4 in CRSwNP pathogenesis and assess its value in discriminating CRSwNP endotypes.

## Materials and methods

### Patient selection and evaluation

A total of 30 CRSsNP patients and 60 CRSwNP patients, who underwent nasal endoscopic surgery between March 2019 and December 2019 in our department, and serum and tissue samples were collected. CRSwNP and CRSsNP were diagnosed following the guidelines of the European Position Paper on Rhinosinusitis and Nasal Polyps 2012 (17). The specific characteristics of CRSwNP in adults are defined as: inflammation of the nose and paranasal sinuses with two or more symptoms, one of which should be nasal blockage/obstruction/congestion or nasal discharge, effusion, facial pain/pressure, decreased or lost sense of smell, and endoscopic bilateral endoscopically visible middle nasal polyps. However, CRSsNP is no visible polyps in the middle meatus, if necessary following decongestant. The exclusion criteria were listed as follows: 1) aged < 18 years or >70 years; 2) fungal sinusitis or acute upper respiratory tract infection; 3) receiving immunotherapy, antibiotics, nasal or systemic corticosteroids, or anti-allergic drugs 1 month before surgery; 4) nasal or sinus malignancy; 5) systemic inflammatory diseases, autoimmune diseases or eosinophilic diseases. VAS, TNSS scores and Lund-Mackay scores were used to assess the severity of the patient's disease. A total of 30 patients undergoing septoplasty for anatomical changes without any sinus disease were enrolled as healthy controls, and uncinate process or middle turbinate tissue was collected during the procedure.

### Diagnosis of CRSwNP endotypes

Nasal polyp tissue was collected during surgery, then the samples were immersed, embedded, sectioned, and stained with hematoxylin and eosin (H & E). Two independent pathologists evaluated the images and counted the number of inflammatory cells, including eosinophils, neutrophils and lymphocytes under 400 x magnification. The diagnosis of CRSwNP endotypes was conducted as previously described (8), tissue specimens were considered eCRSwNP when the percentage of tissue eosinophils (T-EOS) was greater than 10%, otherwise, neCRSwNP was diagnosed.

### Enzyme-linked immunosorbent assay(Elisa)

Peripheral venous blood samples were collected preoperatively from patients, and the samples were stored at room temperature for 1 h after sampling. The collected blood samples were then centrifuged at 3000 rpm at 4 °C, and the supernatant was harvested and stored in aliquots at −80 °C for subsequent experiments. The serum levels of S100A4 were determined using commercially available ELISA kits according to the manufacturer's protocol. All samples were tested in duplicate to improve assay precision.

### Real-Time PCR

Following the manufacturer's protocol, total RNA was extracted from tissue samples using Trizol reagent (Invitrogen). Then reverse transcription of RNA was performed using a commercially available kit (US EVERBRIGHT, Suzhou, China). RT-PCR was performed using PCR system and SYBR Premix EX Taq (US EVERBRIGHT, Suzhou, China), and the following primer sequences were used (Supplementary Material Table S1). Expression of target genes was as a fold increase or decrease relative to GAPDH expression, and the relative mRNA levels of target genes were analyzed by the comparative threshold cycling (2^−*ΔΔ*Ct^) method. The primer sequences are shown in Supplementary Material Table S1.

### Western blot analysis

The polyp tissues and control tissues were homogenized in RIPA buffer containing protease inhibitors and centrifuged at 12,000 rpm for 10 min at 4 °C. The supernatant was collected and protein levels in the extracts were determined using the BCA Protein Assay Kit (New Cell & Molecular Biotech, Suzhou, China). Subsequently, protein samples were electrophoresed on a 15% SDS-PAGE gel and transferred to a PVDF membrane (Millipore). After blocking with 5% skim milk and 0.1% Tween-20 tris-buffered saline (TBST) solution for 1 h at room temperature, rabbit anti-S100A4 antibody (Affinity) was used. The membranes were then washed three times with TBST and incubated with anti-rabbit secondary antibody (Affinity) for 1 h at 37 °C. After extensive washing, the protein bands were observed by enhanced chemiluminescence (UVP, Ltd., United States) according to the manufacturer's instructions.

### Immunofluorescence staining

Immunofluorescence staining was conducted as previously described (18, 19). Tissue specimens obtained during the procedure were immediately fixed in 10% formalin, embedded in paraffin, and sliced into thin sections. Deparaffinized sections (5-*μ*m thick) blocked in goat serum were incubated with the anti-S100A4 antibody (Affinity Biosciences, Changzhou, China). Sections were then washed and incubated with Alexa Fluor 488–conjugated secondary antibody. 4′,6-diamidino-2-phenylindole (Beyotime Biotechnology, Shanghai, China) was used to locate the nucleus. S100A4 expressions in the sections were observed and evaluated by two independent observers and the numbers of positive cells were counted.

### Statistical analysis

4wNormally distributed data were presented as mean ± standard deviation (SD), and non-normal distributed data were described as medians and interquartile ranges (IQRs). For normally distributed variables, one-way analysis of variance (ANOVA) or Student's t-test was performed; otherwise, Kruskal–Wallis H-test or Mann–Whitney *U*-test was applied. Categorical data were expressed as numbers (%) and were compared using the chi-square test. Correlations between S100A4 levels and clinical variables were evaluated by Spearman's correlation analysis. Univariate analysis followed by multivariate logistic regression analysis was conducted to identify the predictive factors associated with CRSwNP endotypes. The receiver operating characteristic (ROC) curve was performed to determine the utility of serum S100A4 and other indications as markers in distinguishing eCRSwNP. A *p*-values <0.05 for two-side was considered as statistically significant.

## Results

### Characteristics of the patients

Table 1 shows the demographic and clinical characteristics of the HCs, CRSsNP, and CRSwNP patients included in this study. There was no significant difference in sex, age, body mass index (BMI), allergic rhinitis, asthma, or smoking among the three groups. However, peripheral eosinophil counts and percentages, VAS scores, and TNSS scores were significantly higher in the CRSwNP group than in the other two groups (all *p* < 0.05). As shown in Table 2, the patients in the Eos CRSwNP group had a significantly higher rate of allergic rhinitis, and peripheral eosinophil percentage than those in the non-Eos CRSwNP group (all *p* < 0.05), but no statistical difference was observed in other variables between two groups (*p* > 0.05).

**Table 1:** The demographic and clinical characteristics among three groups.

Variables	HC (*n* = 30)	CRSsNP (*n* = 30)	CRSwNP (*n* = 60)	*p*-value
Age, years	43.2±11.7	38.7±11.1	39.2±12.1	0.238
Gender (male/female)	17/13	16/14	35/25	0.903
BMI, kg/m^2^	24.9±4.1	25.4±4.5	25.1±4.0	0.912
Allergic rhinitis (yes/no)	0/30	3/27	20/40	0.017
Asthma (yes/no)	0/30	14/16	23/37	0.449
Smoking (yes/no)	9/21	10/20	23/37	0.719
Peripheral eosinophil count, 10^9^/l	0.1±0.09	0.1±0.09	0.2±0.1	<0.001
Peripheral eosinophil percentage, %	0.1±0	0.1±0.1	2.3±0.8	<0.001
VAS score	–	2.5±1.8	5.6±1.9	<0.001
TNSS score	–	4.1±2.8	7.6±2.4	<0.001

HC, healthy control; CRSsNP, chronic rhinosinusitis without nasal polyps; CRSwNP, chronic rhinosinusitis with nasal polyps; BMI, body mass index; VAS, visual analogue scale; TNSS, total nasal symptom scores.

**Table 2:** The demographic and clinical parameters between Eos and non-Eos CRSwNP group.

Variables	Eos CRSwNP group (*n* = 33)	Non-Eos CRSwNP group (*n* = 27)	*p*-value
Age, years	39.1 ± 12.4	39.3 ± 12.0	0.947
Gender (male/female)	20/13	15/12	0.693
BMI, kg/m^2^	25.0 ± 4.2	25.3 ± 3.9	0.777
Allergic rhinitis (yes/no)	16/17	4/23	0.006
Asthma (yes/no)	16/17	7/20	0.074
Smoking (yes/no)	13/20	10/17	0.852
Peripheral eosinophil count, 10^9^/l	0.2 ± 0.1	0.2 ± 0.1	0.899
Peripheral eosinophil percentage, %	2.5 ± 0.7	2.2 ± 0.7	0.029
VAS score	5.5 ± 2.0	5.6 ± 1.9	0.868
TNSS score	7.9 ± 2.7	7.3 ± 1.8	0.317
Lund-Mackay score	15.5 ± 3.8	13.3 ± 3.3	0.024

Eos CRSwNP, eosinophil chronic rhinosinusitis with nasal polyps; BMI, body mass index; VAS, visual analogue scale; TNSS, total nasal symptom scores.

### S100A4 levels in serum and the associations with clinical variables

**Figure 1:**
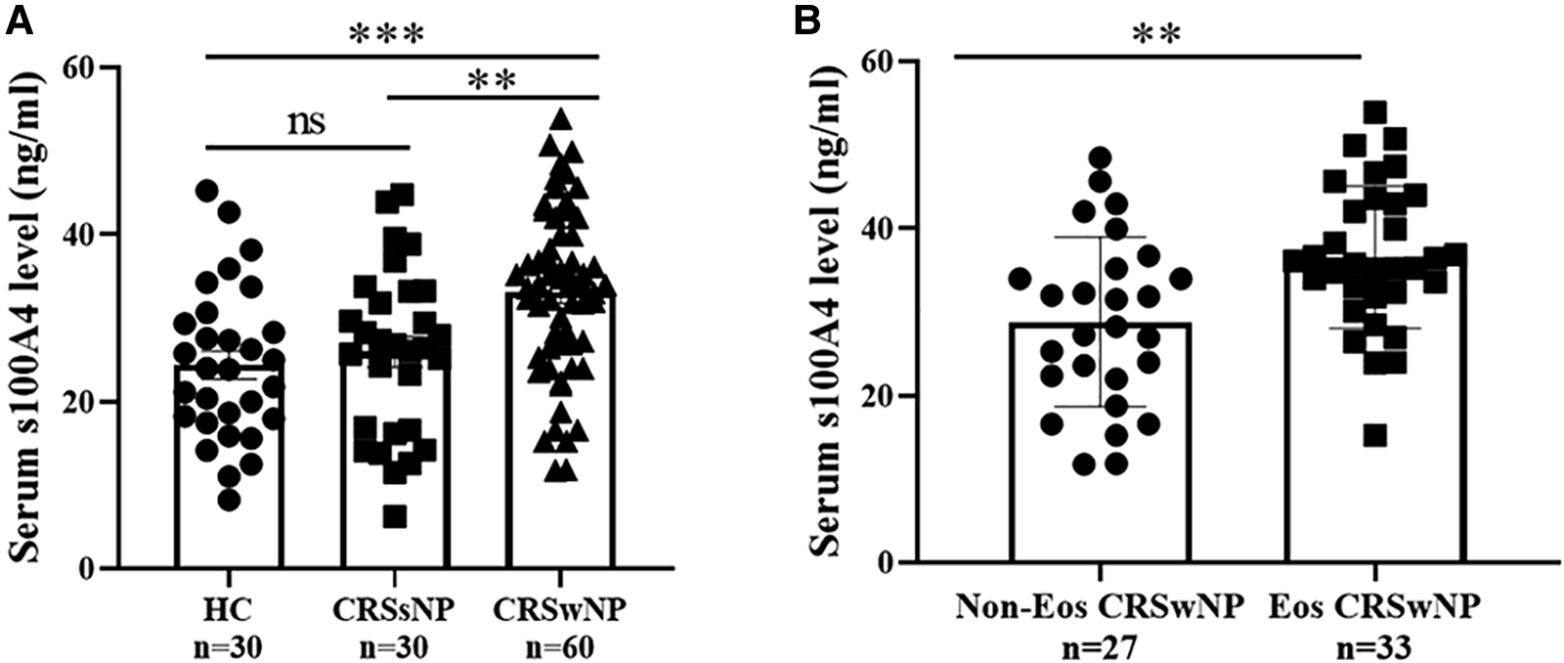
Comparison of serum S100A4 expression among three groups. (**A**) Serum S100A4 levels were increased in the CRSwNP group than the CRSsNP and HC groups. (**B**) Compared to non-EOS CRSwNP, serum S100A4 levels were elevated in the EOS CRSwNP. **p < 0.01, ***p < 0.001, ns, no significance. Abbreviations: CRSwNP, chronic rhinosinusitis with nasal polyps; CRSsNP, chronic rhinosinusitis without nasal polyps; HC, healthy control; EOS CRSwNP, eosinophil chronic rhinosinusitis with nasal polyps.

**Figure 2:**
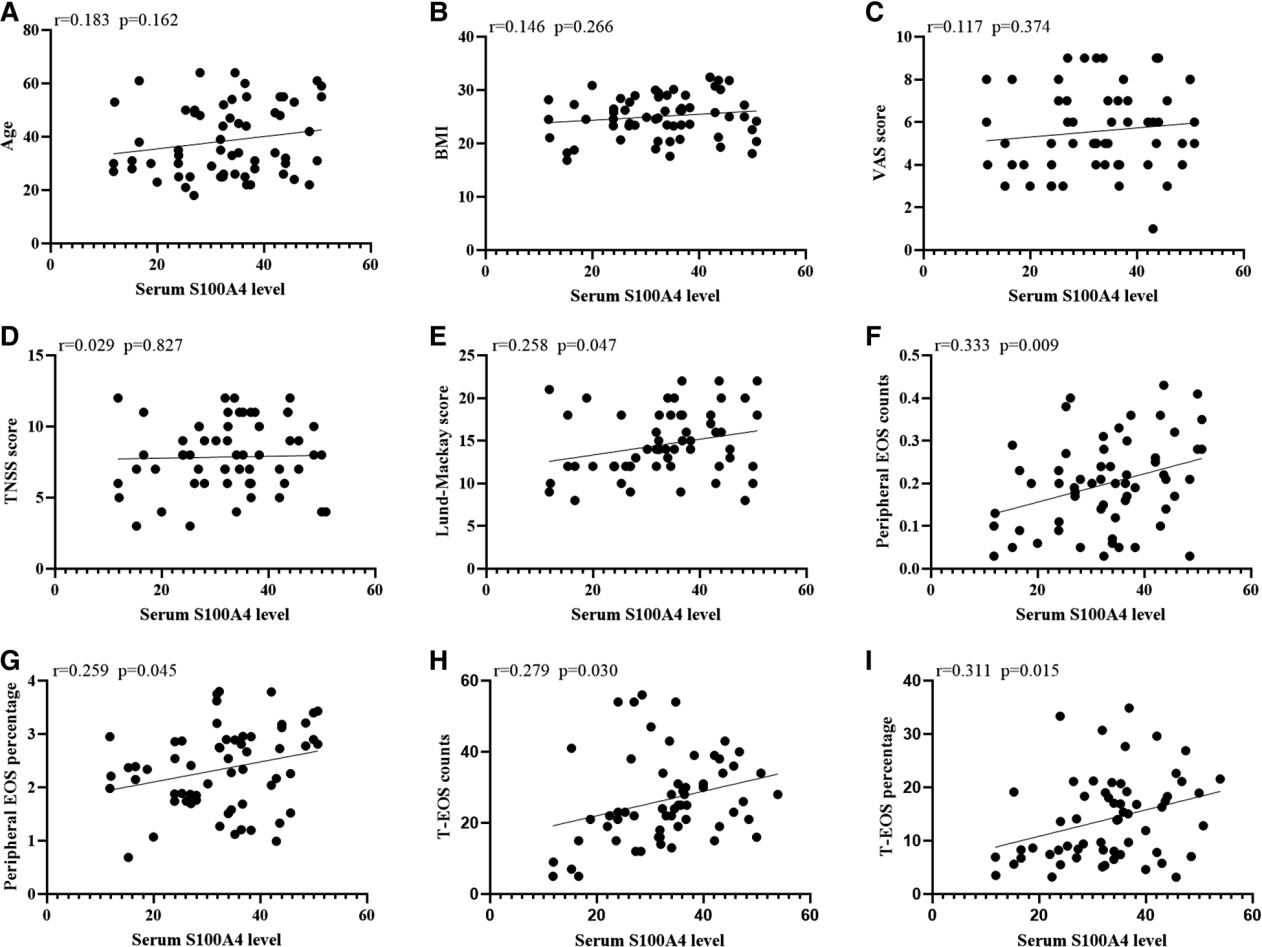
The associations between serum S100A4 levels and clinical variables (**A-I**) in CRSwNP patients. Elevated serum S100A4 mRNA levels exhibited positively correlation with Lund-Mackay score, peripheral EOS count and percentage and T-EOS count and percentage (**E-I**). Abbreviations: CRSwNP, chronic rhinosinusitis with nasal polyps; BMI, body mass index; VAS, visual analog scale; TNSS, total nasal symptom scores; EOS, eosinophil; T-EOS, tissue eosinophil.

The serum S100A4 levels in the CRSwNP group were significantly higher than in the HC group and CRSsNP group, but its levels were not significantly different between the HC group and CRSsNP group (Figure 1A). Moreover, serum S100A4 levels were markedly increased in the Eos-CRSwNP group than in the non-Eos CRSwNP group (*p* < 0.01) (Figure 1B). Moreover, we found that serum S100A4 levels were significantly correlated with Lund-Mackay score (*r* = 0.258 *p* = 0.047), peripheral eosinophil count (*r* = 0.333, *p* = 0.009), and peripheral eosinophil percentage (*r* = 0.259, *p* = 0.045), T-EOS count (*r* = 0.279, *p* = 0.030), and T-EOS percentage (*r* = 0.311, *p* = 0.015) (Figure 2).

## Evaluation of the predictive value of serum S100a4 for CRSwNP endotypes

**Figure 3:**
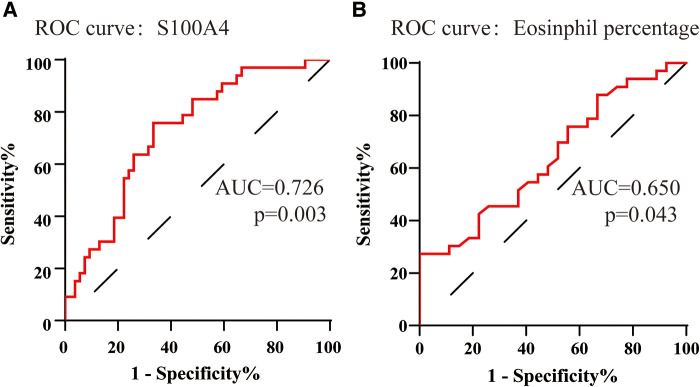
The ROC curves of potentially predictive biomarkers for discriminating EOS CRSwNP. (**A**) serum S100A4; (**B**) peripheral eosinophil percentage. Abbreviations: ROC, receiver operating characteristic; EOS CRSwNP, eosinophilic chronic rhinosinusitis with nasal polyps.

To assess the factors associated with the CRSwNP endotypes, logistic regression analysis was performed. Table 3 showed that blood eosinophil percentages (OR = 2.650, 95% CI = 1.106–6.349, *p* = 0.029) and serum S100A4 (OR = 1.114, 95% CI = 1.033–1.201, *p* = 0.005) were associated with CRSwNP endotypes. The ROC curves in Figure 3 demonstrated that serum S100A4 (AUC = 0.726, *p* = 0.003) exhibited stronger predictive ability for CRSwNP endotypes than blood eosinophil counts (AUC = 0.650, *p* = 0.043). The detailed data were displayed in Table 4.

**Table 3:** Logistic regression analysis of factors affected the rate of recurrence in CRSwNP patients.

Variables	OR	95% CI	*p* value
Age	1.013	0.963–1.065	0.617
BMI	1.031	0.885–1.200	0.698
Peripheral eosinophil count, 10^6^/l	0.999	0.992–1.006	0.703
Peripheral eosinophil percentage, %	2.650	1.106–6.349	0.029
Serum S100A4 level	1.114	1.033–1.201	0.005

CRSwNP, chronic rhinosinusitis with nasal polyps; BMI, body mass index; OR, odds ratio; CI, confidence interval.

**Table 4:** ROC analysis results of different predictors for SCIT efficacy.

Variables	AUC (95% CI)	*p*-value	Cutoff value	Sensitivity	Specificity
Serum S100A4	0.726 (0.595–0.857)	0.003	32.33	0.757	0.667
Peripheral eosinophil percentage	0.650 (0.510–0.790)	0.043	2.06	0.758	0.519

ROC, Receiver operating characteristic; SCIT, subcutaneous immunotherapy; AUC, area under the curve; CI, confidence interval.

## S100A4 expression levels in nasal tissues

**Figure 4:**
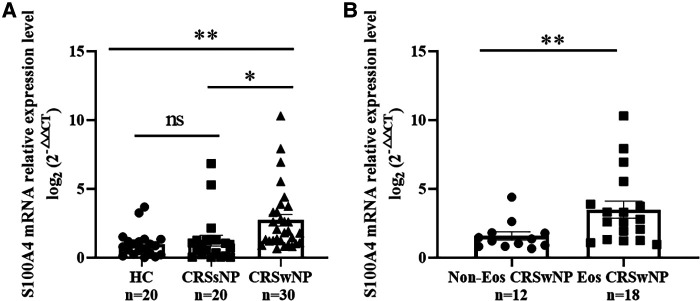
Comparison of S100A4 mRNA levels in the tissue of HC, CRSsNP and CRSwNP patients. (**A**) S100A4 mRNA expressions were elevated in the CRSwNP group than the HC group and the CRSsNP group, but no significant difference was observed between the HC group and the CRSsNP group. (**B**) S100A4 mRNA levels were significantly increased in the EOS CRSwNP group than the non-EOS CRSwNP group. *p < 0.05, **p < 0.01, ns, no significance. Abbreviations: CRSwNP, chronic rhinosinusitis with nasal polyps; HC, healthy control; CRSsNP, chronic rhinosinusitis without nasal polyps; EOS CRSwNP, eosinophilic chronic rhinosinusitis with nasal polyps.

**Figure 5:**
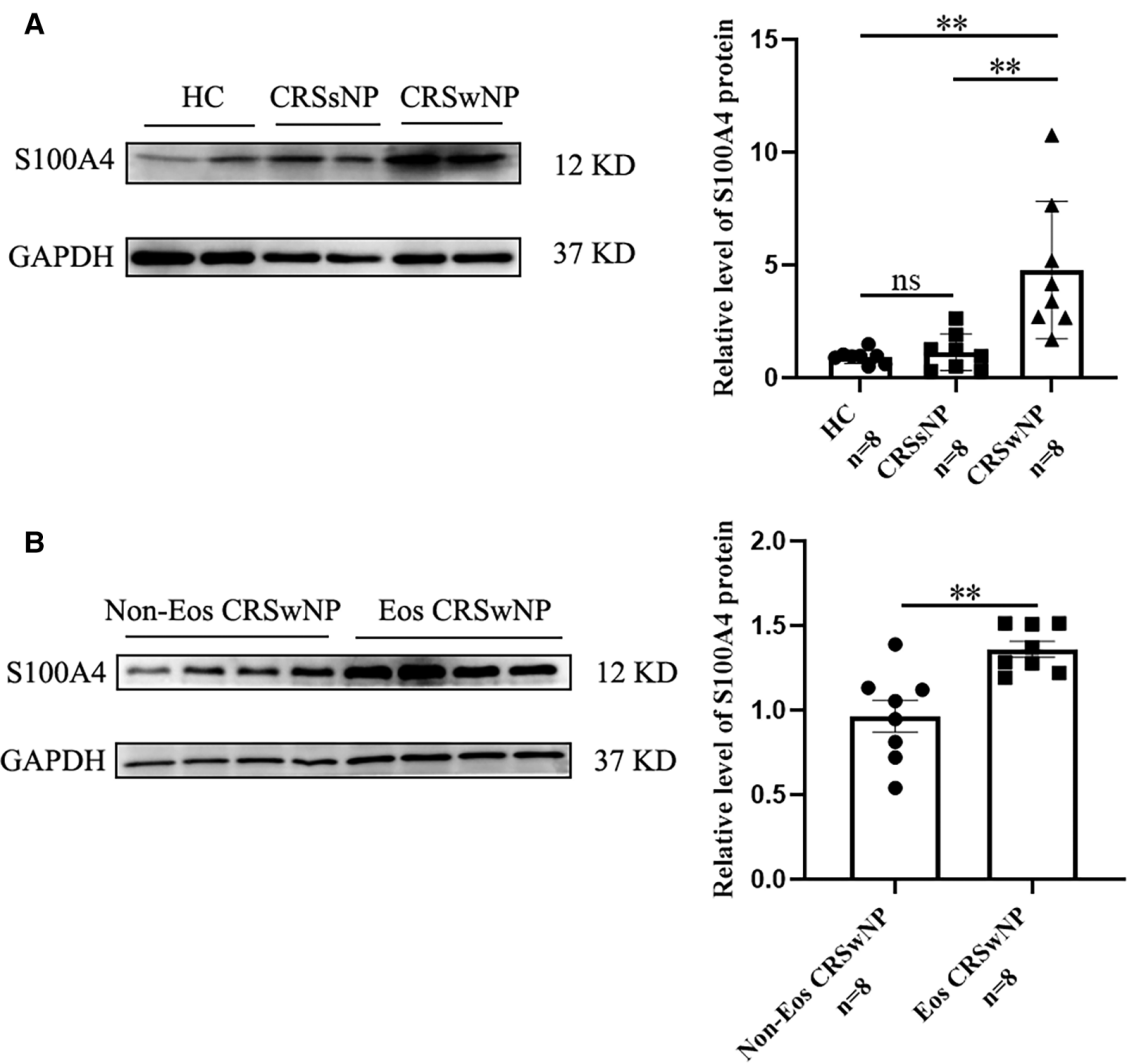
The S100A4 protein expression in the tissue based on western blotting. (**A**) Western blotting images of S100A4 among three groups, and the S100A4 protein relative levels were significantly increased in the CRSwNP group compared to the HC group and the CRSsNP group. (**B**) Western blotting images of S100A4 between the EOS CRSwNP group and non-EOS CRSwNP groups and the S100A4 protein relative levels were significantly higher in the EOS CRSwNP group than non-EOS CRSwNP groups. **p < 0.01, ns, no significance. Abbreviations: CRSwNP, chronic rhinosinusitis with nasal polyps; HC, healthy control; CRSsNP, chronic rhinosinusitis without nasal polyps; EOS CRSwNP, eosinophilic chronic rhinosinusitis with nasal polyps.

**Figure 6:**
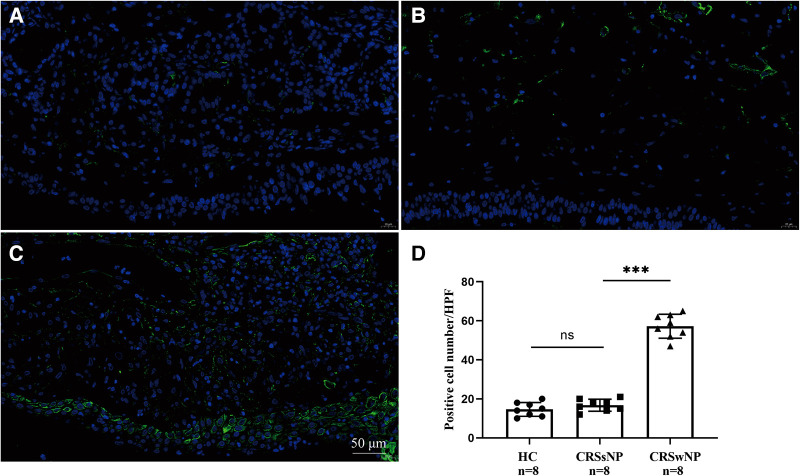
Immunofluorescence and S100A4 protein expression in the tissue indifferent groups. Representative immunofluorescence images of HC (**A**), CRSsNP (**B**) and CRSwNP group (**C**). (**D**) Quantification of S100A4^+^ cells in immunofluorescence staining among three groups. (magnification, x 400) ***p < 0.001, ns, no significance. Abbreviations: CRSwNP, chronic rhinosinusitis with nasal polyps; HC, healthy control; CRSsNP, chronic rhinosinusitis without nasal polyps.

**Figure 7:**
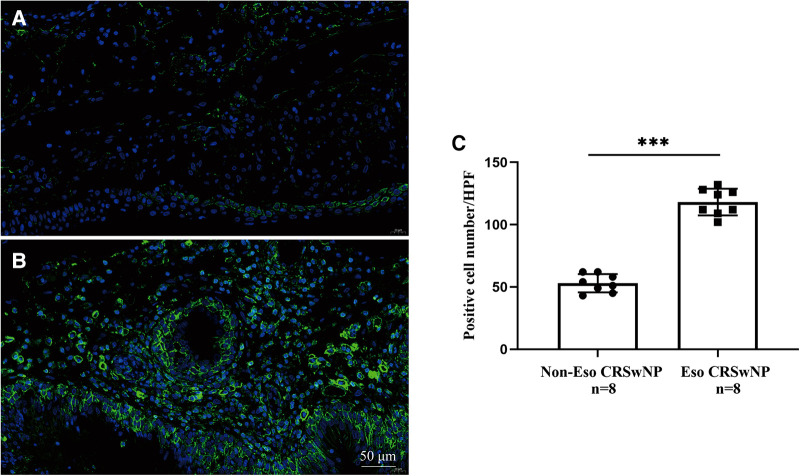
Representative immunofluorescence images of non-EOS CRSwNP (**A**) and EOS CRSwNP patients (**B**). (**C**) Quantification of S100A4^+^ cells in immunofluorescence staining both two groups. (magnification, x 400) ***p < 0.001, ns, no significance. Abbreviations: EOS CRSwNP, eosinophilic chronic rhinosinusitis with nasal polyps.

To further validate the expression levels of S100A4 in nasal tissues, RT-PCR, western blotting and immunofluorescence staining were performed. As illustrated in Figure 4, tissue S100A4 mRNA levels were significantly elevated in the CRSwNP group than in the HC group and CRSsNP group, especially in the Eos CRSwNP group. The western blotting results in Figure 5 indicated that S100A4 protein levels were significantly enhanced in the CRSwNP group, especially in the Eos CRSwNP group. The immunofluorescence staining images showed that the number of protein-positive cells were increased in the CRSwNP group in comparison with HC and CRSsNP groups, and s S100A4taining was mainly located in the nasal epithelial cells and submucosa areas (Figure 6). Moreover, the number of S100A4 positive cells was increased in the Eos CRSwNP group than in the non-Eos CRSwNP group (Figure 7).

## Discussion

To improve the prognosis of CRSwNP patients, increasing attention have been paid to the disease severity and endotypes. Therefore, it is extremely essential to explore objective biomarkers or methods to assess the severity of the disease and differentiate the endotypes of CRSwNP to achieve precise treatment. In the present study, we found that serum and tissue S100A4 levels in the CRSwNP group were significantly higher than in the HC and CRSsNP groups, especially in the Eos CRSwNP group. Moreover, serum S100A4 levels were positively correlated with Lund-Mackay score, circulating and tissue eosinophil counts and percentages, suggesting that its levels were associated with the disease severity and degree of eosinophilic inflammation. In addition, ROC curve analysis indicated that serum S100A4 levels had a predictive value for distinguishing CRSwNP endotypes. Given that, our results demonstrated that S100A4 might be involved in the pathomechanism of CRSwNP and contribute to the development of eosinophilic inflammation, and it might serve as a novel circulating biomarker to evaluate the severity of disease and distinguish endotypes in CRSwNP patients.

S100A4, a pro-inflammatory factor, is secreted by a variety of immune cells and acts as a crucial pro-inflammatory mediator and immune regulator in the inflammatory response (20, 21). Accumulating evidence demonstrated that S100A4 was a candidate risk gene for developing allergic and inflammatory diseases, and its levels were enhanced in the extracellular fluid of these patients (16, 22). In the previous studies, S100A4 levels were observed to be significantly higher in the sputum samples of asthmatics than in healthy controls, and its levels were correlated positively with sputum eosinophils (22). Importantly, stimulation of T cells with S100A4 induces an increase in pro-inflammatory cytokines, particularly eotaxin-2, and they are involved in the pathogenesis of eosinophilic airway inflammation in asthma (23). Moreover, S100A4 was over-expressed in the allergen-induced skin tissue of atopic dermatitis murine models, and the elevated S100A4 levels aggravated the Th2 inflammatory response (16). Šumová et al. (24) found that circulating levels of S100A4 were elevated in patients with axial spondyloarthritis and linked to disease severity. Although S100A4 is considered to be an important pro-inflammatory cytokine, its role in CRSwNP remains poorly understood. Previous study showed that S100A4 was involved in the Th2-type inflammation response and could induce the recruitment of eosinophils. In the present study, we observed that serum and tissue S100A4 levels in CRSwNP patients were significantly elevated than in CRSsNP patients and HCs. Furthermore, the increased S100A4 levels were associated with disease severity and degree of eosinophilic inflammation. Our results are in agreement with most previous results. Therefore, we conclude that S100A4 may act a key role in the pathophysiology of CRSwNP, but the underlying mechanisms are unclear.

Our results also observed that tissue and serum S100A4 levels were significantly higher in the Eos CRSwNP group than in the non-Eos CRSwNP group, and the increased S100A4 levels positively correlated with peripheral and tissue eosinophils count and percentage. Multivariate regression analysis and ROC curve demonstrated that serum S100A4 was a reliable and sensitive indicator for distinguishing Eos CRSwNP from CRSwNP. The underlying mechanisms of Eos CRSwNP have been widely described, and eosinophil inflammation and elevated levels of Th2 cytokines were proven to play key roles in the pathophysiology of Eos CRSwNP (25, 26). Previous publications showed that several immune cells such as macrophages and mast cells were responsible for secreting S100A4, which in turn promote eosinophils to migrate into inflammatory tissues by producing eotaxin and matrix metalloproteinases (23, 27). More importantly, S100A4 could affect T cell differentiation, and lead to a shift of T cells towards Th2 (15, 28). Therefore, we assumed that the inflammatory microenvironment in the nasal polyps prompted the secretion of S100A4 from inflammatory cells, and over-accumulated S100A4 in the tissues would dissociate and enter into peripheral blood as soluble form, resulting in higher serum concentrations of S100A4 in patients with CRSwNP, especially in patients with Eos CRSwNP. The increased local and circulating levels of S100A4 can promote the maturation, proliferation, and migration of eosinophils to a greater extent, and elevated S100A4 enables greater differentiation of T cells toward the Th2 type. This positive feedback loop exacerbates the infiltration of eosinophils into tissues and ultimately aggravates eosinophil and Th2inflammation. Based on the above results, we considered that circulating S100A4 might be involved in the eosinophilic inflammation in the Eos CRSwNP, and serve as an accurate preoperative prediction for CRSwNP endotypes. Thus, our findings provided a clue to quickly distinguish CRSwNP endotypes with a simpler and more accessible biomarker, which contributed to better management and treatment of Eos CRSwNP patients.

However, some shortcomings should be mentioned in the present study. Firstly, the smaller sample size may limit the reliability of the results, and expanded sample size is needed to further confirm our conclusions. Secondly, there are no uniform criteria for the diagnosis of Eos CRSwNP, so an international consensus is urgently needed. Further multicenter studies with uniform diagnostic criteria will confirm and strengthen our conclusions.

In conclusion, our data suggested that S100A4 might participate in the pathogenesis of CRSwNP, and elevated serum S100A4 levels in CSRwNP were associated with disease severity and eosinophilic inflammation. Furthermore, serum S100A4 might be a possible specific biomarker for preoperatively distinguishing CRSwNP endotypes, and contribute to understanding the underlying pathogenesis of Eos CRSwNP. Further studies are needed to determine the role and mechanism of S100A4 in Eos CRSwNP.

## Data Availability

The original contributions presented in the study are included in the article/Supplementary materials, further inquiries can be directed to the corresponding author/s.
