# Supplementary material for: Predictive value of S100A4 in eosinophilic chronic rhinosinusitis with nasal polyps
Source: Front Surg. 2022 Nov 1;9:989489. doi: 10.3389/fsurg.2022.989489 (PMC9663474; doi:10.3389/fsurg.2022.989489)
Supplement: Supplementary file 1 [file Table1.docx]

Table S1 Primer sequences.

| Gene |  | Primers |
| --- | --- | --- |
| GAPDH | Forward | 5’-CTCCTCCTGTTCGACAGTCAGC-3’ |
|  | Reverse | 5’-CCCAATACGACCAAATCCGTT-3’ |
| S100A4 | Forward | 5’-TGGTGAGTTGTGTTGGCCTGACTGG-3’ |
|  | Reverse | 5’-TGCACACCCACGCACACAATCC-3’ |
